# Reducing-agent-free facile preparation of Rh-nanoparticles uniformly anchored on onion-like fullerene for catalytic applications†

**DOI:** 10.1039/c9ra09244g

**Published:** 2020-01-14

**Authors:** Mayakrishnan Gopiraman, Somasundaram Saravanamoorthy, Sana Ullah, Andivelu Ilangovan, Ick Soo Kim, Ill Min Chung

**Affiliations:** Department of Crop Science, College of Sanghur Life Science, Konkuk University Seoul 05029 South Korea illminchung@gmail.com imcim@konkuk.ac.kr +82 02 446 7856 +82 02 450 3730; School of Chemistry, Bharathidasan University Tiruchirappalli 620 024 Tamil Nadu India; Nano Fusion Technology Research Group, Division of Frontier Fibers, Institute for Fiber Engineering (IFES), Interdisciplinary Cluster for Cutting Edge Research (ICCER), Shinshu University Tokida 3-15-1 Ueda Nagano 386-8567 Japan

## Abstract

Herein we report a very simple ‘mix and heat’ synthesis of a very fine Rh-nanoparticle loaded carbon fullerene-C60 nanocatalyst (Rh(0)NPs/Fullerene-C60) for the very first time. The preparation method used no reducing agent and capping agent to control the morphology of the nanocatalyst. Transmission electron microscopy (TEM) results confirmed the uniform decoration of small Rh-nanoparticles on the surface of fullerene-C60. The Rh-content in Rh(0)NPs/Fullerene-C60 was found to be 2.89 wt%. The crystalline properties of Rh(0)NPs/Fullerene-C60 were studied by X-ray diffraction (XRD). The metallic state of Rh-nanoparticles in Rh(0)NPs/Fullerene-C60 was confirmed by X-ray photoemission spectroscopy (XPS). Raman results depicted good interaction between Rh-nanoparticles and fullerene-C60. To our delight, the present Rh(0)NPs/Fullerene-C60 showed excellent catalytic activity in the reduction of 4-nitrophenol with NaBH_4_ in water. Very high *k*_app_, *k*′ and TOF values of 82.14 × 10^−3^ min^−1^, 4107 × 10^−3^ min^−1^ and 138 min^−1^, respectively, were calculated for the Rh(0)NPs/Fullerene-C60 catalyzed reduction of 4-nitrophenol. To the best of our knowledge, this is the most efficient fullerene-based nanocatalyst for the rapid reduction of 4-nitrophenol reported to date. Moreover, the catalytic activity of Rh(0)NPs/Fullerene-C60 was also tested towards Suzuki cross-coupling reactions. Reusability of the Rh(0)NPs/Fullerene-C60 was also tested.

## Abstract 2

Herein we report a very simple ‘mix and heat’ synthesis of a very fine Rh-nanoparticle loaded carbon fullerene-C60 nanocatalyst (Rh(0)NPs/Fullerene-C60) for the very first time.

## Graphical abstract

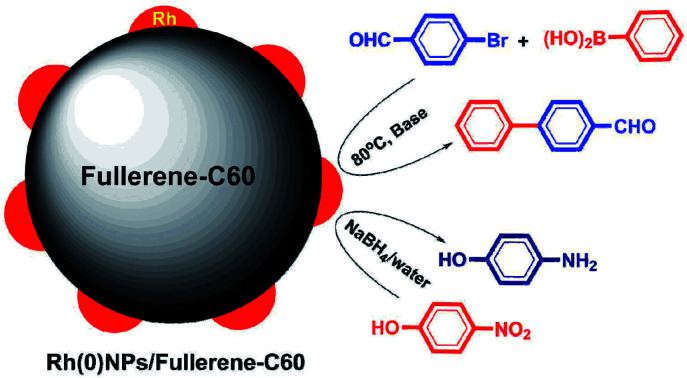

## 1. Introduction

Carbon nanomaterials such as graphene oxide (GO), carbon nanotubes (CNTs), carbon dots (CDs), carbon nanofibers (CNFs), and nano-diamond are demonstrated to be the most suitable platform (support material) for the decoration of catalytic metal-nanoparticles.^1,2^ High activity, easy recovery and reusability of such metal-nanoparticles based carbon nanocomposites are found to be significantly influenced by the nature of support material used.^3^ Indeed, unique structure, fine size, huge surface area and exceptional electrochemical properties make carbon nanocomposites suitable candidates for widespread applications.^4^ In particular, the role of carbon nanocomposites in heterogeneous catalysis is demonstrated to be highly exceptional. For example, Sen *et al.*^5^ prepared thiocarbamide-functionalized graphene oxide (TC@GO) supported Rh/Pt nanocomposites (RhPt/TC@GONPs) *via* a facile method. They found that the RhPt/TC@GONPs is a highly promising catalyst for the Knoevenagel condensation to benzylidenemalononitrile derivatives of aryl aldehydes. Similarly, Gunbatar and co-workers^6^ reported CNTs/Rh-nanoparticles catalyst for hydrolytic dehydrogenation of dimethylamineborane at room temperature. Carbon nanofibers (CNFs) are also widely used as support for the immobilization of active catalytic materials. For instance, Motoyama *et al.*,^7^ decorated Rh-nanoparticles on carbon nanofibers (CNFs) to obtain a highly efficient nanocatalyst (Rh/CNF-T) for arene hydrogenation. The Rh/CNF-T showed high catalytic activity in the hydrogenation of arene under mild conditions in high turnover numbers (TOFs) without leaching of Rh species. Alike, numerous carbon nanocomposites (for example: Fe_2_O_3_/NGr@C,^8^ SWCNT-Met/Pd^0^,^9^ Pt/Au/BDD,^10^ Pd@C-dot,^11^ and Ir/CNFs^12^) are reported as highly efficient catalysts for various industrially important organic reactions. Alike other carbon materials, buckminsterfullerene (carbon fullerene-C60) has also been widely used as key material in diverse applications in chemistry, biology, and nanoscience.^13,14^ The fullerene-C60 incorporated nanosized particles (transition metals or semiconductor) were found to exhibit excellent results in the fields of energy, sensor and catalysis.^15^ For instance, Islam *et al.*,^16^ prepared fullerene-C60 stabilized Au nanoparticles supported on titanium dioxide and used as an efficient catalyst for the degradation of methyl orange and reduction of 4-nitrophenol. Similarly, Ko and co-workers^17^ demonstrated catalytic reduction of 4-nitrophenol using Au-nanoparticle/Fullerene-C60 composites. In spite of that only a limited number of papers published on the carbon fullerene-C60 supported transition metal nanoparticles for catalytic organic conversions. Hence, the development of highly efficient fullerene-C60 based nanocomposites for the catalytic organic reaction is indeed very essential.

Rhodium (Rh) is known for its outstanding catalytic properties, particularly, in hydrogenation, electro-oxidation and coupling reactions.^18–20^ Generally, due to strong resistance to etching of acid/base and heat, the Rh-based catalysts are stable and more versatile in catalytic reaction even under harsh reaction conditions.^21,22^ Hence, significant efforts have been taken for developing simple and efficient wet-chemistry approaches for the preparation of Rh-nanostructures with unique size and shape.^18–23^ Jiang *et al.*,^24^ reported an efficient chemical reduction method for the preparation of mesoporous Rh nanostructures. Recently, Kang and co-workers^25^ obtained three types of Rh-based nanostructures (nanoshells, nanoframes, and porous nanoplates) through an inverse-directional galvanic replacement reaction. However, most of these preparation methods often involve expensive process and toxic reagents. Recently, our group developed a very simple ‘mix and heat’ method for the preparation of various highly active metal-nanoparticles based carbon nanocomposites for organic reaction.^26–30^ We found that the method is not only efficient but also uses no reducing or capping agent to control the size and shape of metal-nanostructures. We believed that the decoration of Rh-nanoparticles on carbon fullerene-C60 would result in highly active nanocomposite for catalytic applications. Herein, highly active Rh-nanoparticles anchored carbon fullerene-C60 nanocomposite (Rh(0)NPs/Fullerene-C60) was successfully prepared by a very simple ‘mix and heat’ method using water. The resultant Rh(0)NPs/Fullerene-C60 catalyst was characterized by means X-ray photoemission spectroscopy (XPS), Raman, X-ray diffraction (XRD), Fourier-transform infrared spectroscopy (FT-IR), scanning electron microscopy with energy dispersive spectroscopy (SEM-EDS), atomic force microscope (AFM) and transmission electron microscopy (TEM). After being characterized, the Rh(0)NPs/Fullerene-C60 was used as catalyst for the reduction of 4-nitrophenol and Suzuki–Miyaura reaction. In fact, both these reactions (reduction and coupling reactions) are very important in organic chemistry and the catalytic products prepared *via* these reactions are found to be highly useful in pharmaceuticals and fine chemicals.^31,32^ Reusability of the Rh(0)NPs/Fullerene-C60 was also studied. Mechanism has also been proposed for the Rh(0)NPs/Fullerene-C60 catalyzed 4-nitrophenol reduction of Suzuki–Miyaura cross coupling reaction.

## 2. Experimental section

### 2.1. Materials

Carbon fullerenes C60 was purchased from Cheap Tubes Inc., United States. Rh-acetylacetonate Rh(acac)_3_, 4-nitrophenol, phenylboronic acid, bromobenzene, 4-bromobenzaldehyde, sodium borohydride (NaBH_4_), base and solvents were purchased from Sigma Aldrich, USA. Double distilled water was used for the sample preparations. All other chemicals were used without further purification.

### 2.2. Preparation of Rh(0)NPs/Fullerene-C60

**Scheme 1:**
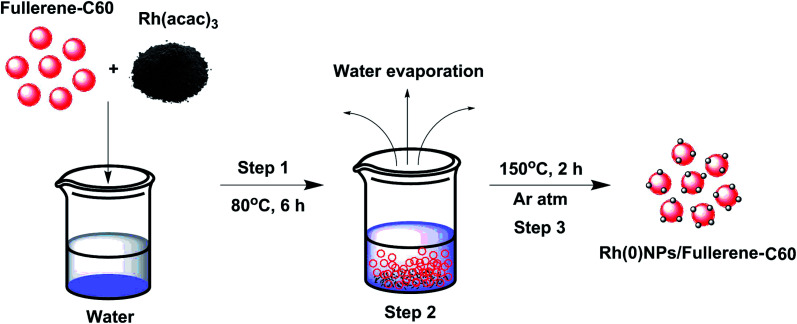
Schematic illustration for the preparation of Rh(0)NPs/Fullerene-C60.

Most of the Rh-nanostructures are produced by wet-chemical synthesis using toxic reducing or capping agents.^18–23^ To our delight, the present preparation method involves no capping or reducing agents. In a typical ‘mix and heat’ preparation of Rh(0)NPs/Fullerene-C60, 500 mg of carbon fullerene-C60 was mixed with 80 mg of Rh(acac)_3_ dissolved in 20 mL of double distilled water and the mixture was stirred at 80 °C for 6 h. Then the water from the above reaction mixture was removed by heating the solution at 100 °C for several hours. The resultant solid mixture of fullerene-C60/Rh(acac)_3_ was mixed well using mortar and pestle for 10 min. Finally, the obtained homogenous mixture of fullerene-C60/Rh(acac)_3_ was calcinated under argon atmosphere at 150 °C for 2 h. The resultant solid catalyst (Rh(0)NPs/Fullerene) was characterized by means of various spectroscopic and microscopic techniques, and used for catalytic applications. Scheme 1 shows the schematic illustration for the preparation of Rh(0)NPs/Fullerene-C60.

### 2.3. Characterization

TEM (JEOL JEM 2100F) instrument was operated at accelerating voltage of 200 kV to record the surface morphology of Rh(0)NPs/Fullerene-C60. Park System model XE100 AFM was used to capture the 1D and 3D AFM images of fullerene-C60 and Rh(0)NPs/Fullerene-C60 in a non-contact mode. Metal loading and elements present in Rh(0)NPs/Fullerene-C60 were determined by EDS analysis (Hitachi 3000H SEM). Crystalline property of samples was studied by using XRD (Rigaku Ultima XRD) and the Raman spectra were recorded on LabRam ARAMIS IR2. Chemical property of the fullerene-C60 and Rh(0)NPs/Fullerene-C60 was investigated by XPS analysis using a Kratos Axis-Ultra DLD, Kratos Analytical Ltd, Japan. FTIR (IR Prestige-21, Shimadzu, Japan) spectra were recorded for both fullerene-C60 and Rh(0)NPs/Fullerene-C60. Catalytic performance of fullerene-C60 and Rh(0)NPs/Fullerene-C60 was studied by UV-vis (Shimadzu UV-2600 spectrophotometer) spectra and GC analysis (Shimadzu-2010 gas chromatograph). NMR (400 MHz Bruker spectrometer) spectra were recorded for the catalytic products.

### 2.4. Procedure for reduction of 4-nitrophenol

Catalytic activity of fullerene-C60 and Rh(0)NPs/Fullerene-C60 was studied towards reduction of 4-nitrophenol. Initially, best reaction conditions were found out. In a typical reduction of 4-nitrophenol, 4 mL of 0.015 M NaBH_4_, 80 μL of 0.01 M 4-nitrophenol and 0.001, 0.0015 or 0.002 mg of Rh(0)NCs/GNPs were sonicated for 15 seconds and the reaction mixture was stirred under room temperature. The progress of the reaction was checked at regular time intervals by using UV-vis spectroscopy.

### 2.5. Suzuki–Miyaura reaction

In a typical procedure, 183 mg of phenylboronic acid (1.5 mmol), 185 mg of 4-bromobenzaldehyde (1.0 mmol), 84 mg of potassium hydroxide and 2.5 mg of Rh(0)NPs/Fullerene-C60 were stirred in 10 mL of 1,4-dioxane/water mixture (8 mL of 1,4-dioxane and 2 mL of water) under N_2_ atmosphere at 80 °C for 24 h. After completion of the reaction, the reaction mixture was analyzed by GC and the catalytic products were extracted using hexane with help of water. Biphenyl (3ab): ^1^H NMR (400 MHz, CDCl_3_): *δ*: 7.58–7.60 (d, 4H), 7.42–7.45 (t, 4H), 7.34–7.37 (t, 2H) ppm; ^13^C NMR (100 MHz, CDCl_3_): d: 141.2, 128.7, 127.5, 127.4 ppm. Biphenyl-4-carbaldehyde (3aa): ^1^H NMR (400 MHz, CDCl_3_) *δ*: 10.02 (s, 1H), 7.93–7.95 (d, 2H), 7.73–7.75 (d, 2H), 7.62–7.64 (d, 2H), 7.47–7.50 (t, 2H), 7.41–7.43 (t, 1H) ppm.

## 3. Results and discussion

### 3.1. Characterization of Rh(0)NPs/Fullerene-C60

**Fig. 1:**
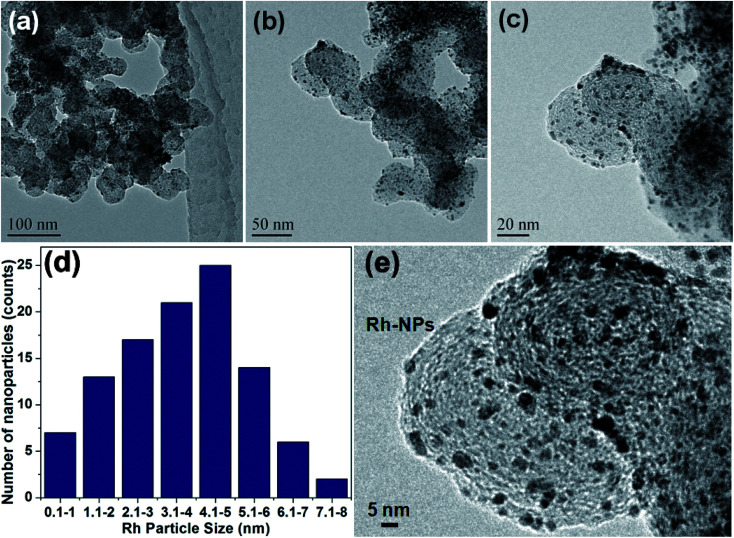
(a–c) TEM images and (e) high resolution TEM images of Rh(0)NPs/Fullerene-C60. (d) Particle size distribution of Rh-nanoparticles in Rh(0)NPs/Fullerene-C60.

**Fig. 2:**
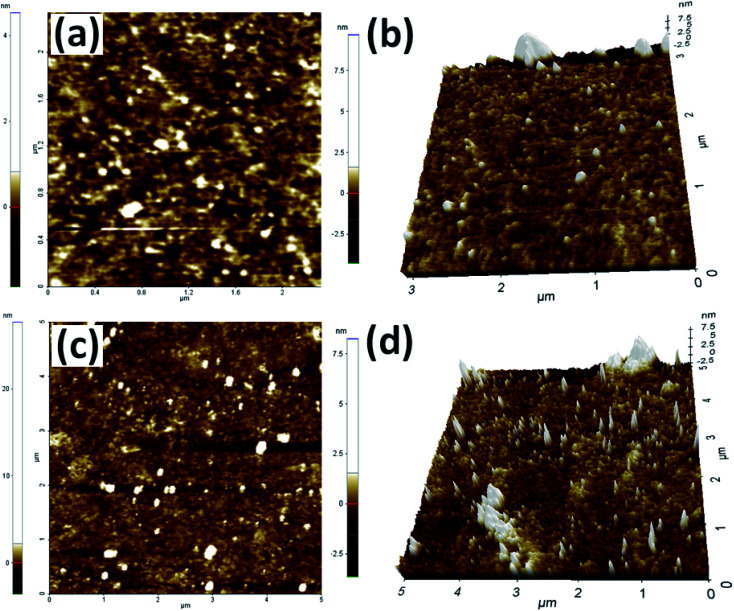
1D and 3D AFM profiles of (a and b) fresh fullerene-C60 and (c and d) Rh(0)NPs/Fullerene-C60.

A simple ‘mix and heat’ method was developed for the preparation of Rh(0)NPs/Fullerene-C60 catalyst by using carbon fullerene C60 and Rh(acac)_3_. A simple calcination of the homogenous mixture of fullerene-C60/Rh(acac)_3_ under inert atmosphere at elevated temperatures successfully produced the present Rh(0)NPs/Fullerene-C60 catalyst. To our delight, water was used to obtain the uniform dispersion of Rh(0)-nanoparticles on the surface of carbon fullerene-C60, and no reducing or capping agent was used. Fig. 1 shows the TEM images of Rh(0)NPs/Fullerene-C60. The size of carbon fullerene-C60 balls varied from 30 to 50 nm. It can be clearly seen from Fig. 1a–c that the onion-like carbon fullerene-C60 ball aggregates consisted of very fine Rh-nanoparticles confirming the efficient loading of Rh-nanoparticles. Surprisingly, the size (mean size ∼4.3 nm) and shape (spherical) of the Rh-nanoparticles were seen to be quite uniform and the Rh-nanoparticles were uniformly dispersed on the surface of fullerene-C60 balls. Although there is no capping agent was used, still the shape and size of the Rh-nanoparticles supported on fullerene-C60 balls could be controlled. Fig. 1e depicts the particle size distribution of Rh-nanoparticles in Rh(0)NPs/Fullerene-C60 which showed that the size of Rh-nanoparticles varied from 0.1 nm to 8 nm with mean size of 4.3 nm. The fresh fullerene-C60 and Rh(0)NPs/Fullerene-C60 were further analyzed by AFM. Fig. 2a–d shows the 1D and 3D AFM profiles of fullerene-C60 before and after Rh(0)-nanoparticles loading. The 1D and 3D AFM profiles of fresh fullerene-C60 showed smooth surface morphology with mean roughness (*R*_q_) values of 91 nm. However, Rh-nanoparticles loaded fullerene-C60 showed *R*_q_ value of 27 nm confirming the high surface roughness due to the uniform decoration of Rh-nanoparticles on the fullerene-C60 surface. Moreover, the mean size of Rh-nanoparticles was calculated to be around 3.9 nm which aggress well with the TEM observations. To further the surface area per unit mass (*S*) of Rh(0)-nanoparticles was calculated by using the equation *S* = 6000/(*ρ* × *d*), where *d* is the mean diameter of Rh(0)NPs (4.3 nm), and *ρ* is the density of Rh (12.41 g cm^−3^).^29^ The *S* value of Rh(0)NPs/Fullerene-C60 was calculated to be 112.44 m^2^g^−1^.

**Fig. 3:**
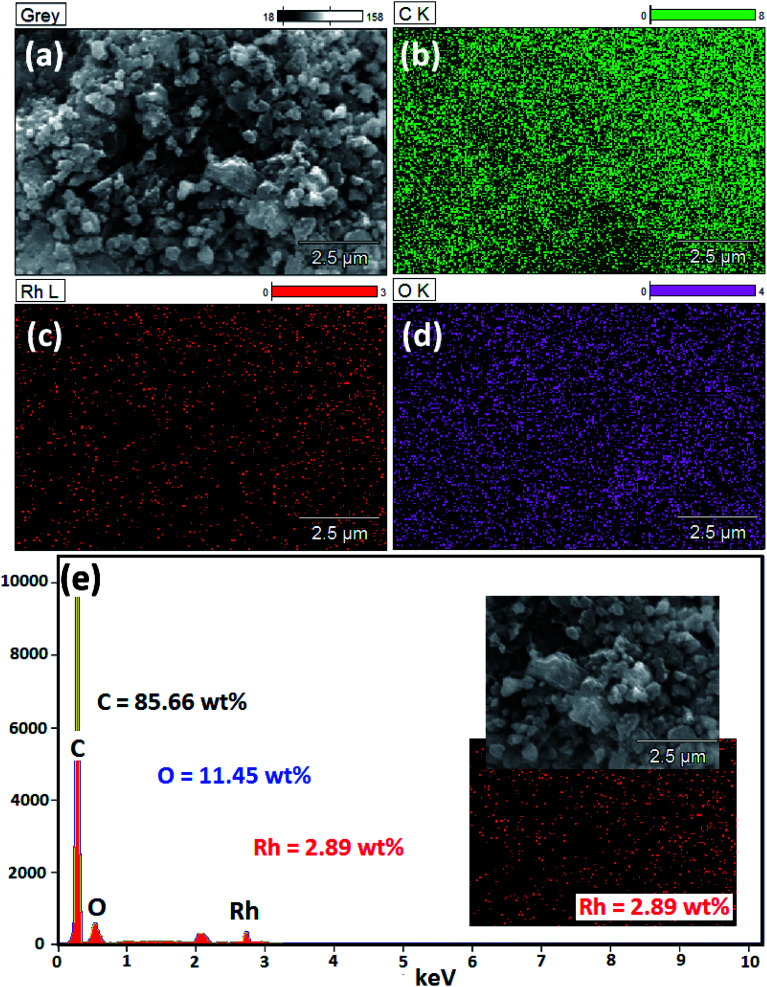
(a) SEM image of Rh(0)NPs/Fullerene-C60 and corresponding (e) EDS spectrum and elemental mapping of (b) C, (c) Rh and (d) O (inset in (e) – SEM and Rh-mapping images).

To further SEM-EDS analysis was performed to find out the elements present in Rh(0)NCs/GNPs catalyst (Fig. 3). The SEM image shows the globular shape of carbon fullerene-C60 ball aggregates consisted of Rh-nanoparticles. Interestingly, the EDS results confirmed the presence of only C, O and Rh elements; indicate the high purity of Rh(0)NCs/GNPs catalyst and reliability of the present preparation method. The loading of Rh in Rh(0)NCs/GNPs was determined to be 2.89 wt%. The EDS wt% of C and O was 85.66 and 11.45, respectively. The elemental mapping of C, O and Rh confirms the homogenous dispersion of Rh-nanoparticles in Rh(0)NCs/GNPs catalyst.

**Fig. 4:**
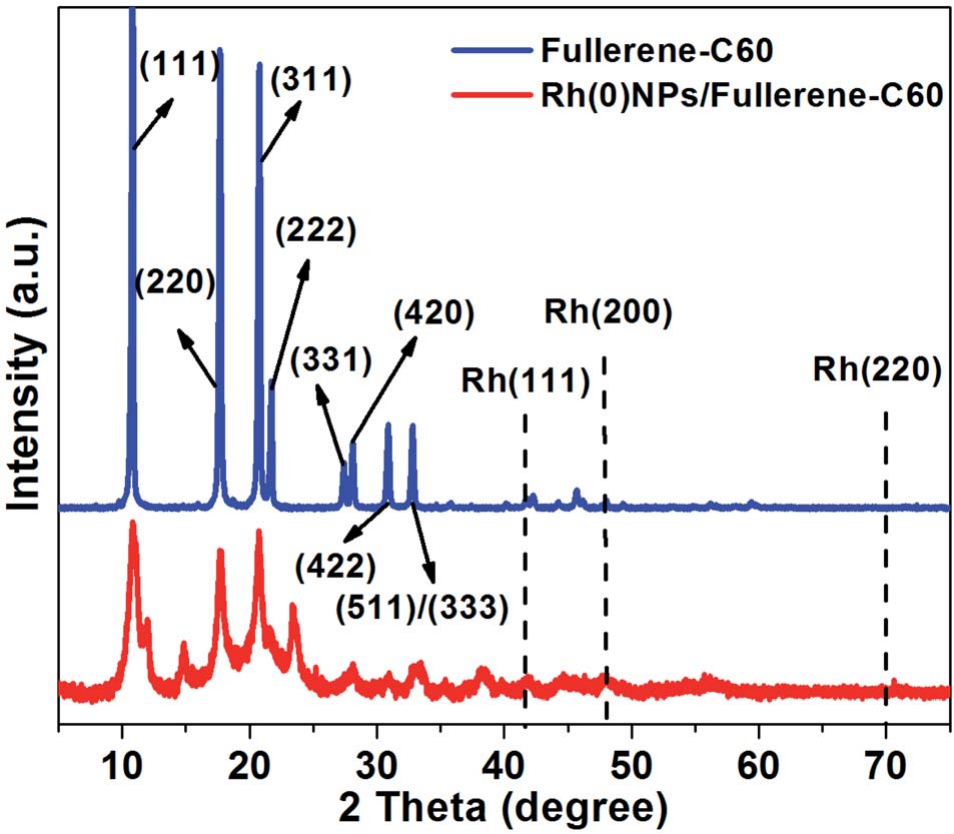
XRD patterns of fresh fullerene-C60 and Rh(0)NPs/Fullerene-C60.

Fig. 4 depicts the XRD patterns of fresh fullerene-C60 and Rh(0)NPs/Fullerene-C60. The fresh fullerene-C60 showed eight characteristic reflections indexed as the (111), (220), (311), (222), (331), (420), (422) and (511)/(333) originate from the fcc lattice of carbon fullerene-C60.^33^ Similarly, the Rh-nanoparticles loaded fullerene-C60 demonstrates three foremost peaks at around 2*θ* = 10.7°, 2*θ* = 17.6°, and 2*θ* = 20.6° which clearly indicate that the loading of Rh-nanoparticles did not significantly alter the crystal structure.^33^ It is believed that the relatively higher electron mobility of fcc structure of the present Rh(0)NPs/Fullerene-C60 would show enhanced catalytic activity. Surprisingly, the XRD pattern of Rh(0)NPs/Fullerene-C60 showed no peaks correspond to the Rh-metal. The absence of Rh-metal peaks is mainly due to the small particle sizes and high degree of dispersion.^26^ Moreover, the peak broadening and overlapping maybe due to the partial structural imperfection or transformation of fcc crystals by the decoration of Rh-nanoparticles on fullerene-C60.

**Fig. 5:**
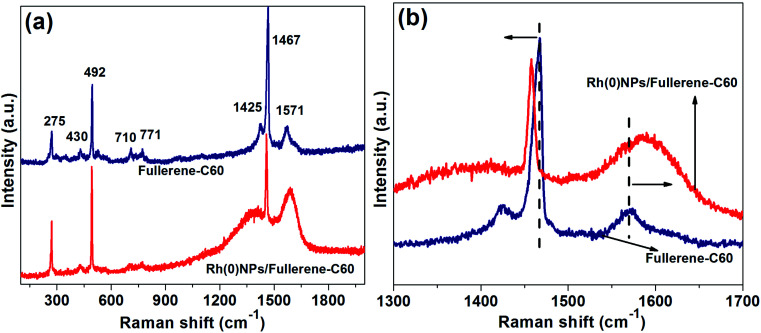
(a and b) Raman spectra of fresh fullerene-C60 and Rh(0)NPs/Fullerene-C60.

The structural characterization of fullerene-C60 before and after Rh-loading was further studied by Raman spectroscopy (Fig. 5). The Raman spectrum of fresh fullerene-C60 showed several lines at 275, 430, 492, 710, 771, 1425, 1467 and 1571 cm^−1^, in which the most intense and significant lines were seen at 492 (A_g_(1) mode) and 1467 cm^−1^ (A_g_(2) mode or ‘pentagonal pinch mode’).^34^ The D band represents the presence of sp^3^ carbons or structural disorder. The G-band at around 1571 cm^−1^ resembles E_2g_ mode of sp^2^-hybridized carbon matrix or graphitic domains. Alike fresh fullerene-C60, the Raman spectrum of Rh(0)NPs/Fullerene-C60 presented several lines with two dominate peaks at 497 and 1452 cm^−1^ correspond to A_g_(1) mode and A_g_(2) mode or ‘pentagonal pinch mode’, respectively. In comparison to fresh fullerene-C60, the Raman lines of Rh(0)NPs/Fullerene-C60 at 497 and 1452 cm^−1^ were found to be shifted to lower frequency (Fig. 5b). In addition, the H_g_(8) line at 1571 cm^−1^ of fresh fullerene-C60 shifted to 1599 cm^−1^ in the Rh(0)NPs/Fullerene-C60 sample. This phenomenon is due to the strong interaction of Rh-nanoparticles with the fullerene-C60.^29^ In general, the covalent interaction of foreign species with fullerene-C60 would often increases the sp^3^-hybridized carbon atoms (increase in the D-band intensity can be seen). In the present case, the Rh-nanoparticles may be physically attached on the surface of fullerene-C60.

**Fig. 6:**
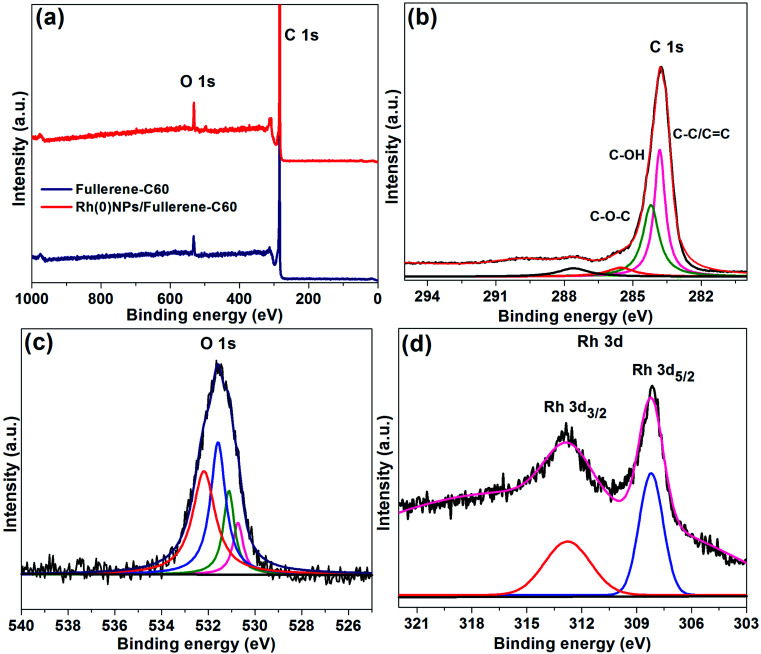
(a) XPS survey spectra of fullerene-C60 and Rh(0)NPs/Fullerene-C60, and deconvoluted (b) C 1s peak, (c) O 1s peak and (d) Rh 3d peaks of Rh(0)NPs/Fullerene-C60.

XPS spectra of fullerene-C60 and Rh(0)NPs/Fullerene-C60 are presented in Fig. 6. It can be seen that both fresh fullerene-C60 and Rh-loaded fullerene-C60 demonstrate two dominate peaks at 283.5 and 531.5 eV correspond to C 1s and O 1s peaks, respectively. The intense C 1s and O 1s peaks clearly show the existence of sp^2^ and sp^3^ carbon along with the surface bound oxygen groups.^27^Fig. 6b and c presents the deconvoluted C 1s and O 1s XPS peaks of Rh(0)NPs/Fullerene-C60. The deconvolation of C 1s peak resulted in four peaks at 283.9, 284.3 and 285.6 eV correspond to C–C/C=C, C–OH and C–O–C groups, respectively.^26,27^ The possible four deconvoluted O 1s peaks (530.8, 531.2, 531.7, 532.5 eV) also confirmed the presence of oxygen groups in the Rh(0)NPs/Fullerene-C60.^30^ In general, due to highly hydrophobic in nature, it is very challenging to achieve good dispersion of fullerene-C60 in aqueous and organic solvents. In the present case, the presence of oxygen groups could assist for the better dispersion of Rh(0)NPs/Fullerene-C60 in a wide range of reaction mediums. In comparison to the fresh fullerene-C60, the XPS spectrum of Rh(0)NPs/Fullerene-C60 showed new peaks at Rh 3d region (321–303 eV), indicating the successful loading of Rh-nanoparticles. Moreover, the Rh 3d_3/2_ peak at 312.8 eV and the Rh 3d_5/2_ peak at 308.3 eV showed the metallic nature of the Rh-nanoparticles.^35^ To further FT-IR spectra were also taken for the fullerene-C60 and Rh(0)NPs/Fullerene-C60 (see Fig. S1 in ESI†). The fresh carbon fullerene-C60 showed a broad peak at 3375 cm^−1^ which obviously confirms the presence of –OH groups. Moreover, three other characteristic peaks at 1570 cm^−1^ (C=C), 1410 cm^−1^ (C–OH), 1175 cm^−1^ and 1040 cm^−1^ (C–O) were also noticed.^36^ In comparison to the fresh carbon fullerene-C60, the FT-IR peak positions of Rh(0)NPs/Fullerene-C60 were noticed to be significantly shifted either to lower or higher frequency region. This phenomenon is may be due to the decoration of Rh-nanoparticles on the fullerens-C60.

### 3.2. Catalytic reduction of 4-nitrophenol

The present Rh(0)NPs/Fullerene-C60 was tested as catalyst for the reduction of 4-nitrophenol in water. The catalytic reduction of 4-nitrophenol to 4-aminophenol is very useful process in green chemistry. In general, the nitrophenols are classified as one of the very common organic pollutants that can be found in environmental wastewater and soil, whereas, the aminophenol is highly useful in the preparation of drugs (such as phenacetin, paracetamol, and so on) and it can be used as reducing agent in dye industry and as photographic developers.^37^ Hence, research on the development of unique and active catalytic material for the conversion of nitrophenol to useful aminophenol is highly deserved. So far, there are several active catalysts were reported for the reduction of 4-nitrophenol to 4-aminophenol. Very recently, Shen *et al.*,^38^ reported catalytic activity of nano-crystalline Au-nanoparticles prepared *via* facile one-pot eco-friendly process. They found that the prepared bio-Ag-nanoparticles are highly efficient in the reduction of 4-nitrophenol to 4-aminophenol. Highly stable hollow nanospheres of metal oxide (NiO, CuO, and NiO/CuO) coated with a porous carbon shell (HNSs@C) were successfully prepared by Wu and coworkers.^39^ The resultant catalysts (HNSs@C) were found be very active in the reduction of 4-nitrophenol with very high *k*_app_ and *k*′ values. Recently, Baran *et al.*, developed highly efficient Pd-based catalysts for the Suzuki–Miyaura reaction and *p*-nitrophenol reduction.^40–47^ Similarly, Ag(0)@chitosan,^48^ Pd-GA/RGO,^49^ CA-AuNPs,^50^ AgNPs/SiNSs,^51^ Ag–P(NIPAM-*co*-AAm),^52^ AgPd–NCs/rGO,^53^ CNC/CTAB/Ag,^54^ AgNPs/PD/PANFP,^55^ Au–Ag@LDH-NPs,^56^ FPP/Au,^57^ and S-graphene (SG)^58^ are also reported. Unfortunately, most of the existing catalytic process show low TOF and *k*′ values which are very important for practical use. To our delight, the present Rh(0)NPs/Fullerene-C60 demonstrated very high *k*_app_ value with excellent TOF (moles of 4-NP converted per mole surface Ni per second) and *k*′ values. Interestingly, carbon fullerene-C60 supported Au nanoparticles catalyst (Au/Fullerene-C60) was prepared and used as catalysts for the reduction of 4-nitrophenol by Ko and co-workers. They found that the Au/Fullerene-C60 is active in the 4-nitrophenol reduction, however, the system not attained complete conversion of 4-nitrophenol to 4-aminophenol even after prolonged reaction time. To the best of our knowledge, this is the first efficient Rh(0)NP-supported fullerene-C60 catalyst for the reduction reaction reported to date.

**Fig. 7:**
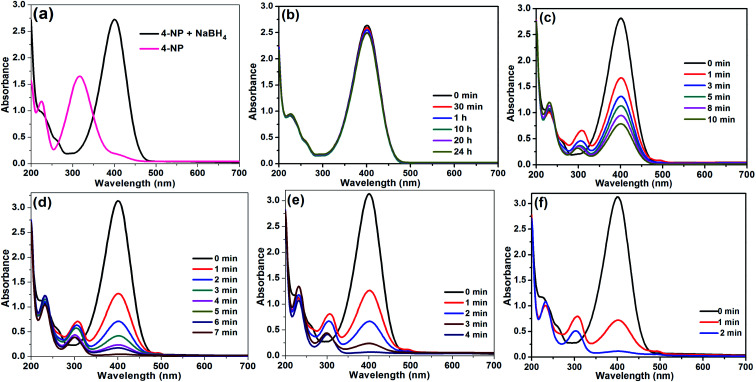
UV-vis spectra: (a) 4-nitrophenol before and after addition of NaBH_4_, (b) GNPs catalyzed reduction of 4-nitrophenol with NaBH_4_, (c–f) reduction of 4-nitrophenol using different amount of Rh(0)NCs/GNPs (0.001, 0.0015, 0.002 and 0.0025 mg).

At first, reaction parameters were optimized. The reactions were carried out in aqueous NaBH_4_ solution. The catalytic performance of Rh(0)NPs/Fullerene-C60 was evaluated by UV-visible spectroscopy (Fig. 7). Fig. 7a shows the UV-vis spectra of 4-nitrophenol before and after the addition of aqueous NaBH_4_. The fresh 4-nitrophenol showed band at 317 nm. After the addition of NaBH_4_, the absorption band at 317 nm red-shifted to 400 nm due to the formation of 4-nitrophenolate ions. Based on the results, 80 μL of 0.01 M 4-nitrophenol, and 4 mL of 0.015 M aqueous NaBH_4_ were opted as optimal reaction conditions. In order to check the catalytic activity of fresh carbon fullerene-C60, 0.1 mg of carbon fullerene-C60 was also used as catalyst for the reduction of 4-nitrophenol (Fig. 7b). As expected, the carbon fullerene-C60 was found to be inactive in the reduction reaction. The stirring reaction mixture with fresh carbon fullerene-C60 as catalyst showed no change in the adsorption maximum at 400 nm even after 24 h. The results confirm that the fresh carbon fullerene-C60 is inactive in the reduction process and also free from metal impurities. To our delight, the presence of Rh(0)NPs/Fullerene-C60 rapidly reduces 4-nitrophenol to 4-aminophenol with NaBH_4_. Initially, 1 mg of Rh(0)NPs/Fullerene-C60 was used as catalyst, however, the system converted 4-nitrophenol in less than 10 seconds. Then the amount of Rh(0)NPs/Fullerene-C60 was gradually reduced to 0.001–0.0025 mg. To our delight, very small amount of Rh(0)NPs/Fullerene-C60 (0.0015, 0.002 and 0.0025 mg) was found to be enough for the complete reduction of 4-nitrophenol (Fig. 7d–f). The 0.001 mg of Rh(0)NPs/Fullerene-C60 is not enough for the complete reduction of 4-nitrophenol (Fig. 7c). It can be seen that the 0.0025 mg of Rh(0)NPs/Fullerene-C60 showed rapid reduction of 4-nitrophenol. Surprisingly, the 0.0025 mg of Rh(0)NPs/Fullerene-C60 took only 2 min for the 100% conversion of 4-nitrophenol to 4-aminophenol. To the best of our knowledge, this is the most efficient catalyst reported for the rapid reduction of 4-nitrophenol.

**Fig. 8:**
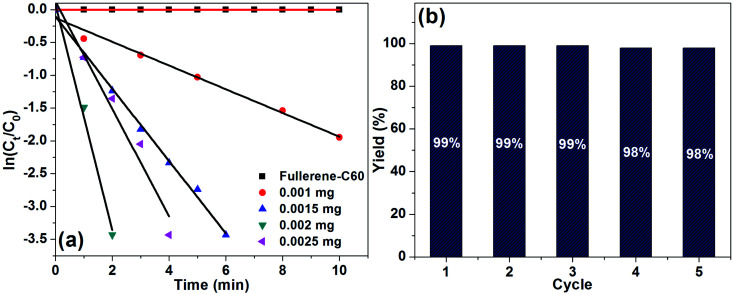
(a) Plots of ln[*C*_*t*_/*C*_0_] *versus* reaction time for the reduction of 4-nitrophenol with NaBH_4_ over different amounts of Rh(0)NCs/GNPs, and (b) reusability of Rh(0)NCs/GNPs.

Reaction kinetics was studied for the Rh(0)NPs/Fullerene-C60 catalyzed reduction of 4-nitrophenol. In the present case, the role of fresh carbon fullerene-C60 and the concentration of NaBH_4_ were not considered for the kinetic studies. In fact, the carbon fullerene-C60 is not active in the reduction process and an excess amount of NaBH_4_ was used. Fig. 8a shows plots of ln[*C*_*t*_/*C*_0_] *versus* reaction time for the reduction of 4-nitrophenol with NaBH_4_ over different amounts of Rh(0)NCs/GNPs. The relationship between ln(*C*_*t*_/*C*_0_) and time was noticed to be liner with good correlation coefficient (*R*^2^ of about 0.9971) which shows that the Rh(0)NPs/Fullerene-C60 catalyzed reduction of 4-nitrophenol follows pseudo-first-order reaction kinetics. From the slop of the liner plots, the kinetic reaction rate constant (*k*_app_) was calculated. Form the *k*_app_ values, reaction rate constant per unit mass (*k*′) values were calculated using the formula; *k*′ = *k*/*M*, where *M* – weight of the metal active site added. Moreover, turn over frequency (TOF, moles of 4-nitrophenol converted per mole surface Rh per minutes) was also calculated. Surprisingly, the *k*_app_, *k*′ and TOF values are found to be very high when compare to previous results (see Table 1). The *k*_app_ values for the Rh(0)NPs/Fullerene-C60 catalyzed conversion of 4-nitrophenol to 4-aminophenol were calculated to be 10.75 × 10^−3^ min^−1^ (0.001 mg), 16.18 × 10^−3^ min^−1^ (0.0015 mg), 82.14 × 10^−3^ min^−1^ (0.002 mg) and 132.17 × 10^−3^ min^−1^ (0.0025 mg). The excellent *k*′ value of 4107 × 10^−3^ mg^−1^ min^−1^ was calculated for the 0.0025 mg of Rh(0)NPs/Fullerene-C60 catalyzed reduction of 4-nitrophenol. Surprisingly, the present system achieved very high TOF number of 138 min^−1^. The reusability of Rh(0)NPs/Fullerene-C60 was also tested in the reduction reaction (Fig. 8b). The Rh(0)NPs/Fullerene-C60 was found to be reusable for several runs without loss in its catalytic ability. The present catalyst showed about 98% conversion of 4-nitrophenol to 4-aminophenol even at its 5th use.

**Table 1:** Comparison of present Rh(0)NPs/Fullerene-C60 over other previously reported catalysts^a^

S. no	Catalyst	*k*_app_ (×10^−3^ s^−1^)	*k*′ (×10^−3^ mg^−1^ s^−1^)	TOF (s^−1^)	References
1	Ni/GNS	3.06	1.53	0.31	35
2	NiO/CuO@C	25.1	—	—	39
3	Pd-GA/RGO	1.99	1.99	—	49
4	Rh(0)NPs/Fullerene-C60	2.2	68.5	2.3	This work
5	RhAg0.5/rGO	14.8	1415	—	49
6	AgPd NCs/rGO	0.365	0.365	—	53
7	AgNPs/PD/PANFP	2.283	—	—	55
8	Au–Ag@LDH-NPs	0.375	—	—	56
9	FPP/Au	3.75	3.75	—	57
10	Au–Ag/r-GO (0.1)	3.47	34.7	0.042	60
11	Ru/HHP	62.1	31.1	—	61
12	Ni/CNFs	69.1	27.6	0.03	62
13	Cu/C	0.3	0.13	0.15	63
14	Ru/C	1.3	0.52	0.29	63
15	Ni-oxide/GOSs	60.8	60.8	0.73	64

a TOF, s^−1^: (turnover frequency) moles of 4-NP converted per mole surface Ni per second.

The activity of Rh(0)NPs/Fullerene-C60 was compared with previously reported results (Table 1). To the best of our knowledge, *k*_app_, *k*′ and TOF values are found to be very high when compare to previous results (see Table 1). For example, the NiO-nanoparticles supported carbon nanocomposite (NiO/CNP) demonstrated good catalytic activity toward reduction of 4-nitrophenol with *k*′ value of 1.53 × 10^−3^ mg^−1^ s^−1^ whereas the present Rh(0)NPs/Fullerene-C60 showed better *k*′ value of 6.9 × 10^−2^ mg^−1^ s^−1^.^35^ Meng *et al.*,^59^ reported MoS_2_/reduced graphene oxide (1T-MoS_2_/RGO) nanocomposite for the reduction of 4-nitrophenol. The 1T-MoS_2_/RGO nanocomposite showed *k*′ value of 0.011 × 10^−3^ min^−1^ g^−1^ whereas the present catalyst demonstrated better *k*′ value. Hareesh and co-workers prepared Ag–Au-rGO nanocomposite and it was used for the reduction of 4-nitrophenol. The Ag–Au-rGO nanocomposite showed *k*_app_ and TOF values of 34.7 × 10^−3^ s^−1^ and 2.5 min^−1^, respectively. To our delight, the present catalytic system showed *k*_app_ and TOF values of 132.17 × 10^−3^ min^−1^ and 138 min^−1^, respectively. Similarly, transition metal nanoparticles immobilized human hair or cellulose nanofibers supports showed good catalytic activity in the reduction of 4-nitrophenol whereas the present Rh(0)NPs/Fullerene-C60 showed better activity. Table 1 shows the comparison of present Rh(0)NPs/Fullerene-C60 over other reported catalysts. The catalytic activity of Rh(0)NPs/Fullerene-C60 can be compared with the other reported catalysts.

**Scheme 2:**
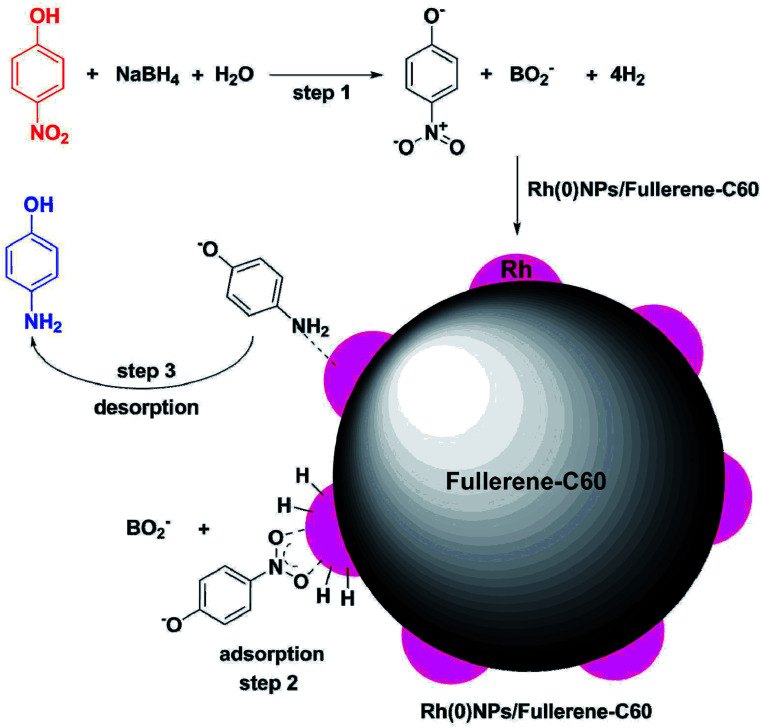
Proposed mechanism for Rh(0)NPs/Fullerene-C60 catalyzed reduction of 4-nitrophenol to 4-aminophenol in the presence of NaBH_4_.

Scheme 2 demonstrates the proposed mechanism for the Rh(0)NPs/Fullerene-C60 catalyzed reduction of 4-nitrophenol to 4-aminophenol with NaBH_4_. Stirring the aqueous mixture of 4-nitrophenol, NaBH_4_ and Rh(0)NPs/Fullerene-C60 leads to adsorption of nitrophenolate ion (acceptor group) on the surface of Rh(0)NPs/Fullerene-C60 and the donor BH^4−^ produces active hydrogen atoms on the catalyst surface (as shown in Scheme 2). Subsequently, the formed active hydrogen atoms reduces the adsorbed 4-nitrophenolate ion to 4-aminophenol. Finally, the product 4-aminophenol desorbs from the surface of Rh(0)NPs/Fullerene-C60. The result shows that the Rh(0)NPs/Fullerene-C60 reduces 4-nitrophenol in very short reaction time of just 2 min. This is mainly due to the presence of Rh-nanoparticles with small size in Rh(0)NPs/Fullerene-C60. In addition, the carbon fullerene-C60 might have assisted for the rapid transfer of electrons from BH_4_^−^ to the Rh surface in Rh(0)NPs/Fullerene-C60.

### 3.3. Suzuki cross-coupling reaction

Indeed, Suzuki cross-coupling reactions are the prime protocols for the carbon–carbon construction.^65^ Particularly, the synthesis of biaryl compounds *via* Suzuki cross-coupling has become one of the most preferred methods in organic chemistry.^40–47,66–72^ In fact, this reaction is workable with wide range of substrates under mild reaction conditions, and the reaction starting material (boronic acid) is highly sustainable and readily available. Moreover, the catalytic products, biaryl compounds, are vastly useful in the production of various pharmaceuticals, liquid crystals, complex drug molecules, ligands and polymers.^65,66^ So far, various transition metals-based homogenous and heterogeneous catalysts are developed for the Suzuki cross-coupling reactions. Among them the heterogamous systems are most preferred due to easy recovery and reusability of the catalysts. Most of the heterogeneous catalysts developed for Suzuki cross-coupling reactions are Pd-based systems. For example, Erami *et al.*,^67^ prepared Pd nanoparticles supported G-COOH catalysts for the Suzuki–Miyaura C–C coupling reactions. They found that the resultant Pd-catalyst is highly active, versatile, stable and reusable. Similarly, magnetically separable nanocatalyst (Pd-nanoparticles supported on the surface-modified Fe_3_O_4_/SiO_2_ nanoparticles) was reported for Suzuki and Heck cross-coupling reactions by Du and co-workers.^68^ Other transition metals such as Co, Ni, Cu, Rh, Ru, Ag, Pt and Au are also reported as efficient catalysts for the Suzuki cross-coupling reactions. For instance, Han *et al.*,^69^ reported facile preparation of stable Au nanoparticles and their catalytic activity toward Suzuki–Miyaura cross-coupling reaction in water. Alike, boron nitride nanosheet-anchored Pd–Fe core–shell nanoparticles was prepared and used as nanocatalysts for Suzuki–Miyaura coupling reactions by Fu and co-workers.^70^

The supported Rh-nanoparticles are demonstrated to be one of the most highly active catalysts for various organic transformations. Gniewek *et al.*,^72^ reported Rh(0) nanoparticles supported PVP catalyst (Rh/PVP) for Suzuki–Miyaura reaction and hydrogenation of benzene. Unfortunately, the Rh/PVP showed moderate activity towards the Suzuki–Miyaura reaction. Later, Wang *et al.*,^70^ prepared bimetallic Pd–Rh nanocatalyst with tunable morphologies and compositions. The obtained bimetallic Pd–Rh nanocatalyst was found to be highly active toward Suzuki cross-coupling reactions. However, the preparation of bimetallic Pd–Rh nanocatalyst required toxic reducing and capping agent and halide anions (Br^−^/I^−^) as shape control agents. To our delight, the present mono metallic Rh(0)NPs/Fullerene-C60 was found to be highly active for the Suzuki–Miyaura reaction. Surprisingly, no reducing or capping agents were used to control the particle size of Rh.

**Scheme 3:**
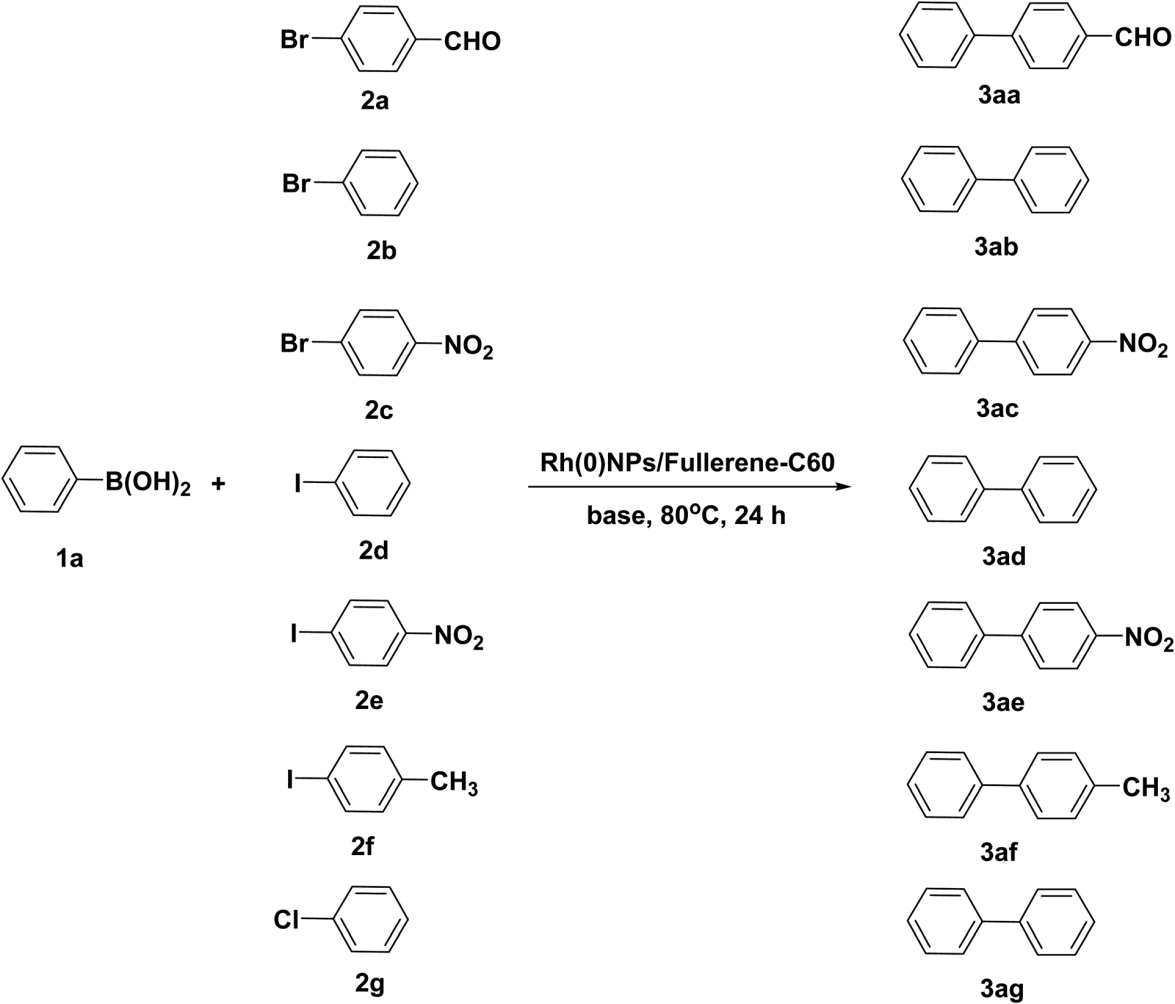
Rh(0)NPs/Fullerene-C60 catalyzed Suzuki cross-coupling reaction.

Scheme 3 shows the Rh(0)NPs/Fullerene-C60 catalyzed Suzuki cross-coupling of phenylboronic acid (1a) with various aryl halides. Initially, reaction parameters such as catalyst amount, reaction time, temperature, base and solvent were screened to find out the most suitable reaction conditions. The coupling of phenylboronic acid (1a) with 4-bromobenzaldehyde (2a) was taken as model reaction. As expected, trace amount of the desired product, biphenyl-4-carbaldehyde (3aa), was obtained in the absence of catalyst. Alike, the fresh carbon fullerene-C60 was also found to be inactive in the cross-coupling reaction. The reaction solvent (1,4-dioxane/water mixture (8 mL of 1,4-dioxane and 2 mL of water)) and time (24 h) were opted as best reaction conditions. Four different amounts of Rh(0)NPs/Fullerene-C60 (1, 2, 2.5 and 3 mg) was used and found that 2.5 mg is enough to achieve the maximum yield of 3aa. Alike, KOH, NaOH and K_2_CO_3_ were used as base for the cross-coupling reaction. Among them, KOH was found to be the best one. Reaction temperature was found to play crucial role in the yield of the product. The Rh(0)NPs/Fullerene-C60 gave about 12% of 3aa when the reaction was stirred at room temperature. The optimal reaction temperature was found to be 80 °C. Under the optimized reaction conditions, the scope of the catalytic system was extended. The Rh(0)NPs/Fullerene-C60 gave better yield of 87% of the desired product (3aa) whereas Rh/PVP catalyst afford 65% of the desired product. Alike, Rh(0)NPs/Fullerene-C60 catalyzed cross-coupling of 1a and 2b afford biphenyl (3ab) in good yield of 85%. Coupling reaction between 1-bromo-4-nitrobenzene (2c) and phenylboronic acid (1a) yielded 89% of the 4-nitro-1,1′-biphenyl (3ac). Similarly, iodobenzene (2d) and its derivatives were also allowed to react with phenylboronic acid (1a) in the presence of Rh(0)NPs/Fullerene-C60. Under the optimized reaction conditions, the reaction between 1a and iodobenzene (2d) gave the desired product (3aa) in excellent 92% yield. The derivatives of iodobenzene, 1-iodo-4-nitrobenzene (2e) and 1-iodo-4-methylbenzene (2f), were also reacted with phenylboronic acid (1a) to give 4-nitro-1,1′-biphenyl (3ae, 83%) and 4-methyl-1,1′-biphenyl (3af, 61%), respectively. Surprisingly, less reactive chlorobenzene (2g) was allowed to react with 1a to obtain the desired product, biphenyl (3ag). However, the present system showed poor yield of 37% under the optimized reaction conditions. It was found that the Rh(0)NPs/Fullerene-C60 can be reused at least for 4 cycles without significant loss in its catalytic activity. At 4th cycle, the Rh(0)NPs/Fullerene-C60 gave 73% of the 3aa from the cross-coupling reaction between 1a and 2a. The catalytic performance of the Rh(0)NPs/Fullerene-C60 can be compared with previously reported catalysts such as Pd/Fe_3_O_4_/SiO_2_,^68^ Pd–Fe,^70^ Rh/PVP,^72^ and Pd–Rh nanocatalyst.^71^

**Scheme 4:**
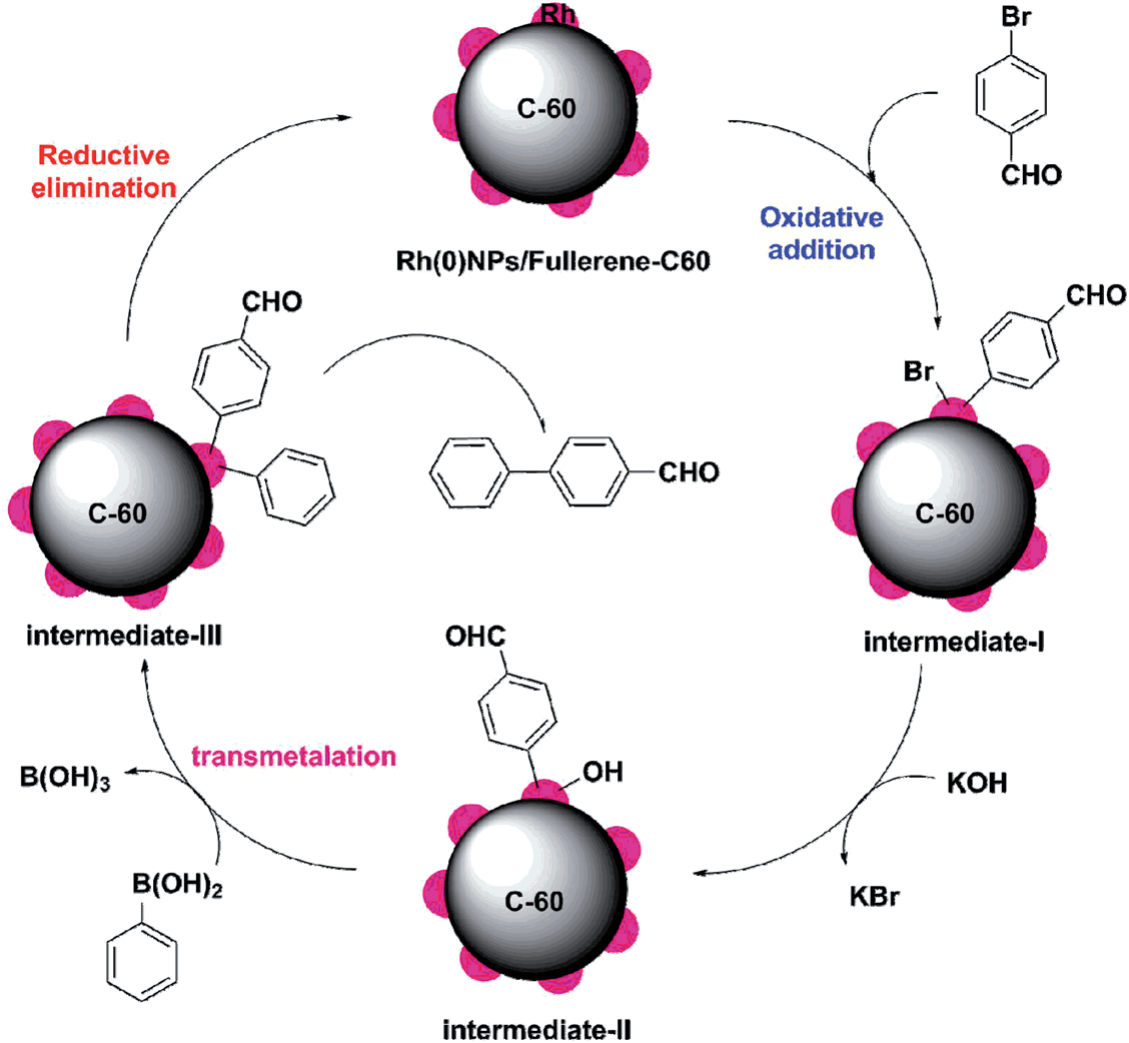
Proposed mechanism for the Rh(0)NPs/Fullerene-C60 catalyzed Suzuki–Miyaura reaction of phenylboronic acid with 4-bromobenzaldehyde.

Scheme 4 depicts the proposed mechanism for the Rh(0)NPs/Fullerene-C60 catalyzed Suzuki–Miyaura reaction of phenylboronic acid with 4-bromobenzaldehyde. The mechanism involves three main steps: oxidative addition, transmetallation and reductive elimination. At first, the aryl halide species adsorbs on to Rh(0)NPs/Fullerene-C60 through oxidative addition and forms intermediate-I. Subsequently, transmetallation step occurs by the conversion of intermediate-I in the presence of base to nucleophilic Rh alkoxy complex (intermediate-II). Then the intermediate-II reacts with neutral organoboron compound and form intermediate-III (diaryl complex). Finally, the reductive elimination of intermediate-III gives biaryl derivative.

The present Rh(0)NPs/Fullerene-C60 catalyst is found to be efficient in both reduction of 4-nitrophenol and Suzuki–Miyaura reaction for several reasons: (i) small size of Rh-nanoparticles, (ii) good attachment of Rh-nanoparticles with carbon fullerene-C60, (iii) good dispersion of Rh(0)NPs/Fullerene-C60 in aqueous and organic solvents (due to the presence of small amount of oxygen functional groups) and (iv) high Rh-nanoparticles surface area. In addition, the simple and green preparation method also advantage of this present work.

## 4. Conclusions

Highly active Rh(0)NPs/Fullerene-C60 catalyst was successfully obtained by a simple ‘mix and heat’ method for the very first time. To our delight, the preparation method involved just water without using any reducing or capping agents. Rh-nanoparticles with mean size of 4.3 nm were uniformly dispersed on the surface of fullerene-C60. The Rh-content in Rh(0)NPs/Fullerene-C60 was determined to be 2.89 wt%. The chemical state of Rh-nanoparticles was 0 (metallic Rh), as confirmed by XPS. The crystalline property of Rh(0)NPs/Fullerene-C60 and, good interaction between Rh-nanoparticles and fullerene-C60 were confirmed. The present Rh(0)NPs/Fullerene-C60 exhibited *k*_app_, *k*′ and TOF values of 82.14 × 10^−3^ min^−1^, 4107 × 10^−3^ min^−1^ and 138 min^−1^, respectively, towards the reduction of 4-nitrophenol with NaBH_4_ in water. To the best of our knowledge, this is most efficient catalyst for the rapid reduction of 4-nitrophenol reported to date. The utility of the present Rh(0)NPs/Fullerene-C60 towards Suzuki cross-coupling reaction with a variety of functionalized substrates was also demonstrated. The Rh(0)NPs/Fullerene-C60 demonstrated excellent catalytic activity in Suzuki cross-coupling reaction. Reusability of the Rh(0)NPs/Fullerene-C60 was also found to be good.

## Author contributions

Conceptualization and methodology, M. G., I. S. K., and I. M. C.; formal analysis, M. G., and S. S.; software and formal analysis, S. U., and M. G.; data curation and investigation, M. G. and S. S.; original draft writing and review and editing, M. G., I. M. C. and I. S. K.; supervision, A. I., I. M. C. and I. S. K.

## Funding

This research received no external funding.

## Conflicts of interest

Authors declare there is no conflict of interest.

## Supplementary Material

RA-010-C9RA09244G-s001
