# Supplementary material for: Reducing-agent-free facile preparation of Rh-nanoparticles uniformly anchored on onion-like fullerene for catalytic applications
Source: RSC Adv. 2020 Jan 14;10(5):2545–59. doi: 10.1039/c9ra09244g (PMC9048634; doi:10.1039/c9ra09244g)
Supplement: RA-010-C9RA09244G-s001 [file RA-010-C9RA09244G-s001.pdf]

Electronic Supplementary Material (ESI) for RSC Advances.
This journal is © The Royal Society of Chemistry 2020

Supporting Information

Reducing-agent-free facile preparation of Rh-nanoparticles uniformly anchored on onion-like fullerene for catalytic applications

Mayakrishnan Gopiraman,[1] Somasundaram Saravanamoorthy,[2] Sana Ullah,[3] Andivelu Ilangovan,[2] Ick Soo Kim,[3] Ill Min Chung[1,*]

[1]*Department of Applied Bioscience, College of Life & Environment Science, Konkuk University, 120 Neungdong-ro, Gwangjin-gu, Seoul 05029, South Korea*

[2]*School of Chemistry, Bharathidasan University, Tiruchirappalli, 620 024, Tamil Nadu, India*

[3]*Nano Fusion Technology Research Group, Division of Frontier Fibers, Institute for Fiber Engineering (IFES), Interdisciplinary Cluster for Cutting Edge Research (ICCER), Shinshu University, Tokida 3-15-1, Ueda, Nagano Prefecture 386-8567, Japan*

*Corresponding author. Tel.: +82 02 450 3730; fax: +82 02 446 7856.

E-mail addresses: illminchung@gmail.com (I.M. Chung).

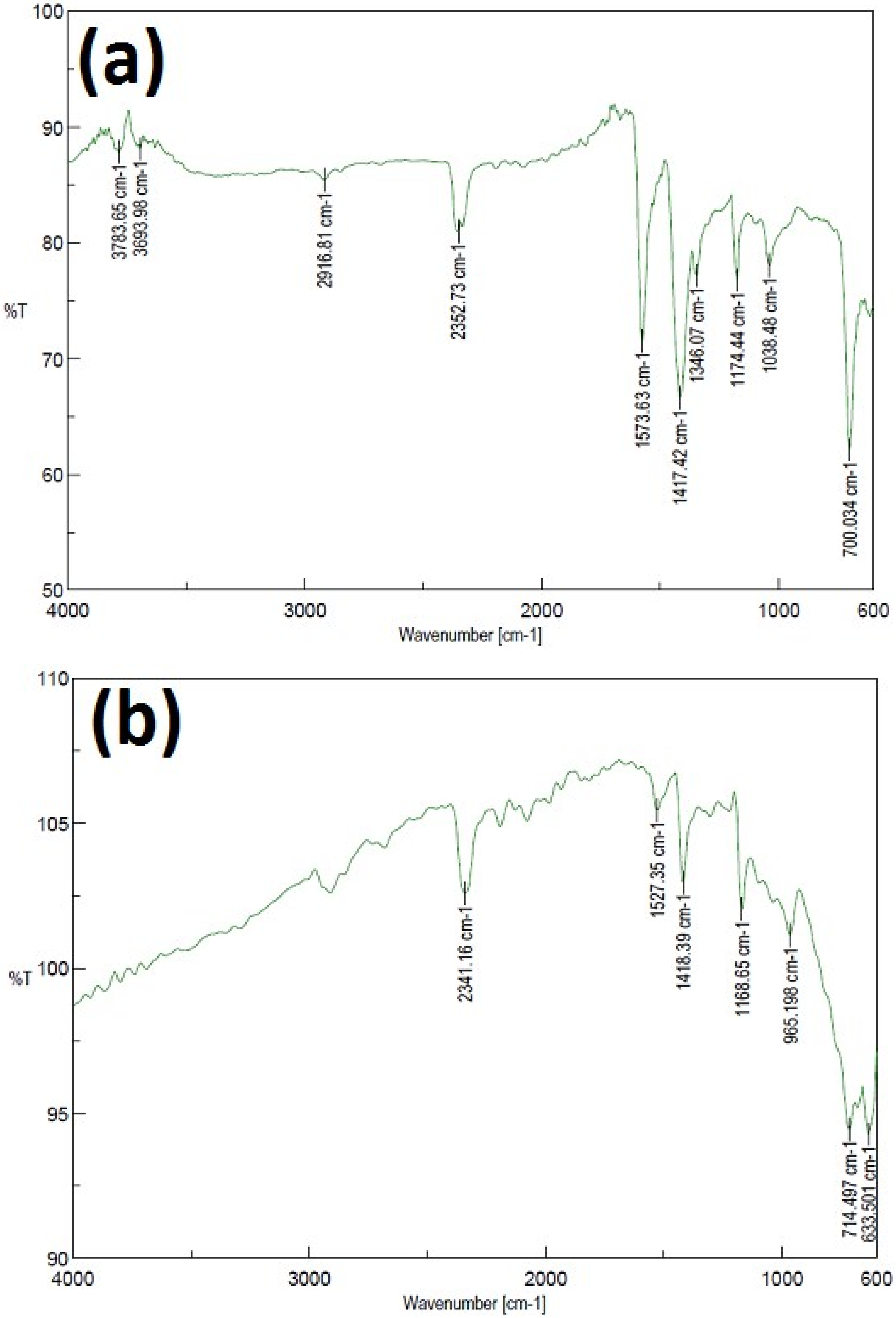

Fig. S1. FT-IR spectra of (a) carbon Fullerene-C60 and (b) Rh(0)NPs/Fullerene-C60.

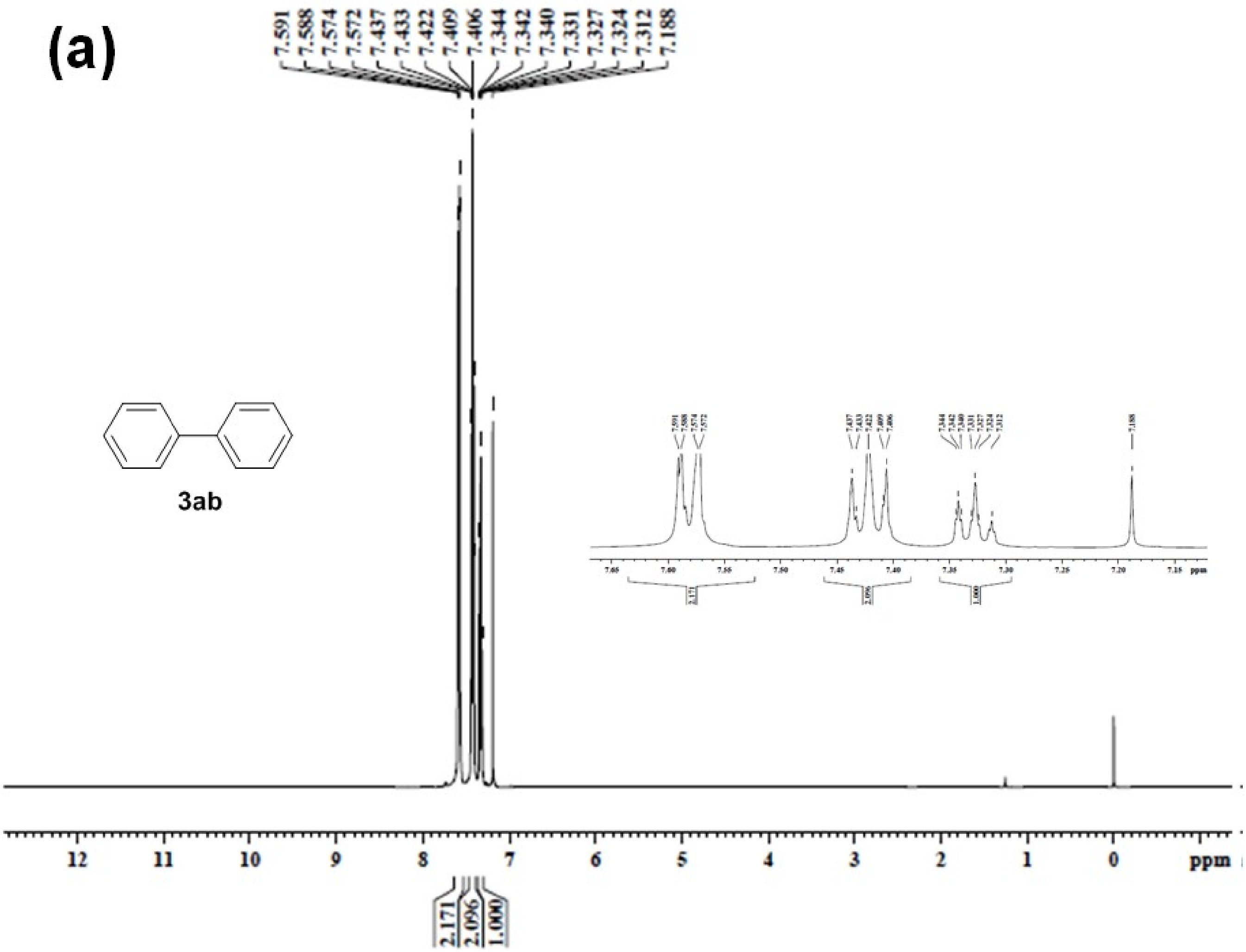

Fig. S2. ^{1}H NMR spectra of biphenyl (**3ab**).

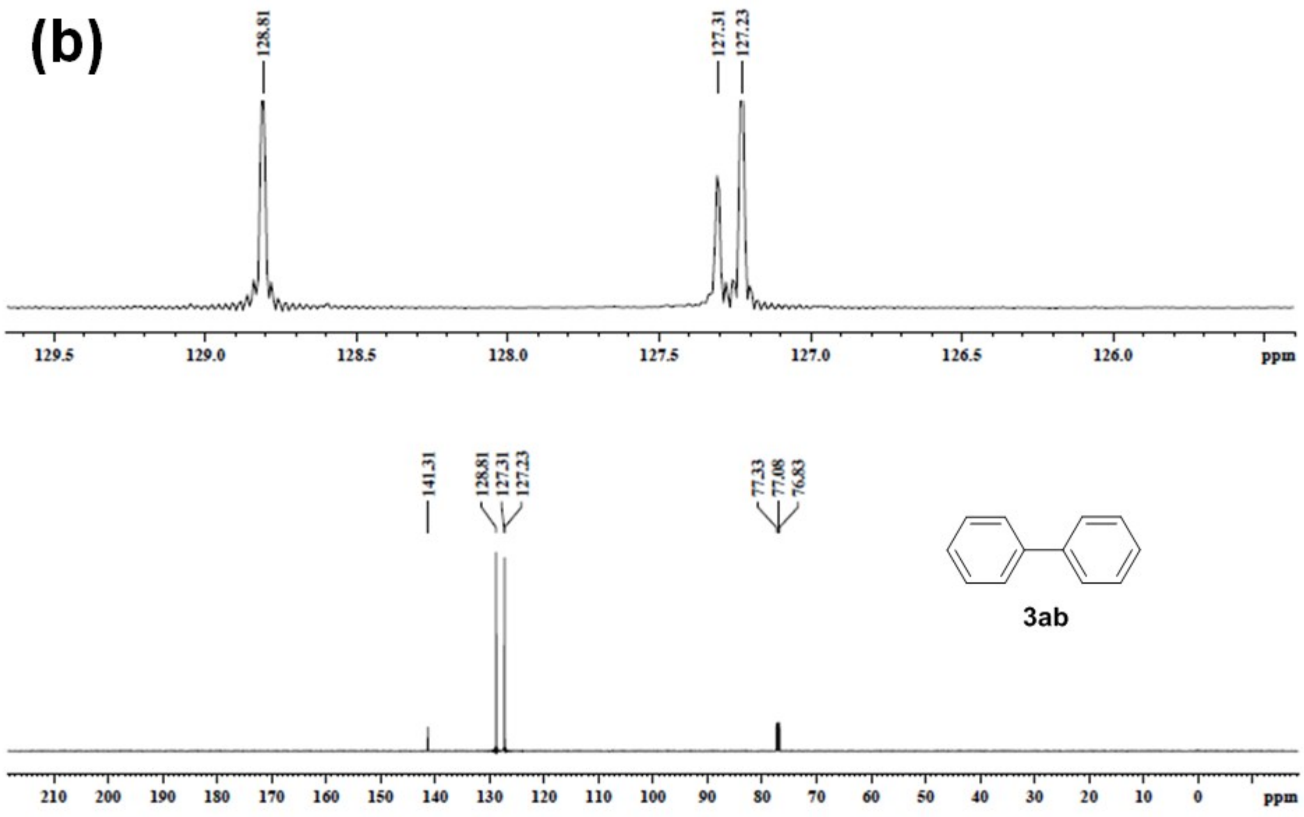

Fig. S3. ^{13}C NMR spectra of biphenyl (**3ab**).
